# Breast conserving therapy for central breast cancer in the United States

**DOI:** 10.1186/s12893-022-01488-0

**Published:** 2022-01-29

**Authors:** Jiameng Liu, Xiaobin Zheng, Shunguo Lin, Hui Han, Chunsen Xu

**Affiliations:** 1 The Graduate School of Fujian Medical University, Fuzhou, 350000 Fujian China; 2 Department of Breast Surgery, Fujian Medical University Union Hospital, No. 29, Xinquan Road, Fuzhou, 350001 Fujian China; 3 Department of General Surgery, Fujian Medical University Union Hospital, Fuzhou, 350001 Fujian China; 4 Breast Cancer Institute, Fujian Medical University, Fuzhou, 350001 Fujian China; 5 Department of Radiotherapy, Fujian Medical University Cancer Hospital, Fuzhou, 350000 Fujian China

**Keywords:** Central breast cancer, Nipple-areola complex, Breast conserving therapy, Overall survival, Breast cancer-specific survival

## Abstract

**Introduction:**

Although central breast cancer is not a contraindication to breast conserving, most surgeons still choose to perform total mastectomy. The safety of breast conserving treatment for central breast cancer is still unclear. The purpose of this study is to evaluate the long-term survival outcome of central breast cancer.

**Materials and methods:**

Using SEER database to explore the trend of surgical procedures for patients with central breast cancer. The patients were divided into breast conserving group and non-breast conserving group. Multivariate logistic regression was used to evaluate predictors of breast conserving surgery in central breast cancer. The clinicopathological variables were adjusted through the multivariable Cox risk model, and the stage and T stage were stratified to compare survival results.

**Results:**

A total of 8702 patients with central breast cancer underwent surgical treatment from 2010 to 2015. There were 3870 patients in the breast conserving group and 4832 patients in the non-breast conserving group. The breast preservation rate was 44.4%, which rose from 39.9% in 2010 to 51% in 2015. Elderly patients (*p* < 0.001) and low tumor malignancy were predictors of breast conserving therapy. In the 1:1 matched case–control analysis, breast cancer-specific survival (BCSS) (*p* < 0.001) and overall survival (OS) (*p* < 0.001) in breast conserving therapy group were still higher than those of non-breast conserving. In the subgroup analysis of T staging and stage, the breast conserving therapy group still had higher OS and BCSS.

**Conclusion:**

In central breast cancer, breast-conserving therapy is safe and optional.

## Introduction

Breast conserving therapy (BCT) allows patients to achieve esthetic outcomes, quality of life and preserve their breast without sacrificing oncologic outcome [1–3] and is considered as a safe treatment for early-stage breast cancer.

Central breast cancer usually refers to tumors located in the area within 2 cm of the nipple-areola complex (NAC). The research on BCT of central breast cancer were few and small sample size though the results showed acceptable recurrence rate of BCT in central breast cancer (4.8–7%) [4–6] and the non-inferior survival outcomes [5, 7, 8] compared with non-BCT. So for central cancers breast conserving therapy was not contraindication in the guideline, but was less likely to be recommended by surgeons for reasons below: (1) careful pathologic examination of mastectomy specimens has found that more than 30% involve the nipple-areola complex [9–11] and lumpectomies that remove the nipple-areola complex often result in poor cosmesis. (2) Perceived increase in the risk of local recurrence owing to inadequate margins. Recent stunning result was reported from a SEER data based research including 16522 central breast cancer which showed an improved survival rate for centrally located breast cancer (CLBC) receiving BCT [12]. But the early studies on the safety of BCT for CLBC [4, 13–16] or the comparation of oncological outcomes between BCT and non-BCT [7, 8] and the recent SEER based result [12] were all constrained to T1-2 stage without taking T3-4 into account which cannot meet the increasing demand for more cosmetically acceptable breast cancer surgery. Also HER-2 status was an important factors influencing the survival outcome of breast cancer, which was not included in the recent SEER based result. So a study on the survival difference between BCT and non-BCT in central and NAC, especially in T3-4 subgroup population is urgently need.

## Materials and methods

### Data source and study population

The Surveillance, Epidemiology, and End Results (SEER) database was used to evaluate the safety of breast conserving therapy. We acquired permission to download and analyze data for academic purpose (reference number: 10727-Nov2020). This study does not contain any experiments on humans as well as animals and/or the use of human tissue samples performed by any of the authors. The SEER cancer registries provide population-based cancer surveillance for 17 areas that represent approximately 26% of the United States. Inclusion criteria: (1) the diagnosing year ranged from 2010 to 2015, (2) the primary site of tumor was breast, (3) tumor site was central portion of breast (C50.1) or nipple (C50.0), and (4) patients underwent breast surgery. Exclusion criteria: (1) patients with stage IV disease, (2) patients with unknown information of race, diagnosing year, marital status or important clinicopathological data, (3) patients younger than 18 years old or elder than 80, (4) patients with a history of other cancer, (5) patients with less than 1 month survival after diagnosis, and (6) patient’s diagnoses were only depended on biopsy or autopsy. Finally, a total of 8702 adult breast cancer patients aged 19 to 79 years between 2010 and 2015 was included, and we stratified patients into 2 groups by type of surgery: breast conserving therapy (n = 3870) and non-breast conserving therapy (n = 4832). The non-breast conserving therapy included mastectomy and breast reconstruction.

### Statistical analysis

Chi-squared testing was used to compare the differences in baseline characteristics between patients treated with non-BCT versus patients treated with BCT. Multivariable logistic regression was used to identify factors associated with surgery type. Kaplan–Meier analysis was used to compare overall survival outcomes between patients treated with different surgery type. Univariate and Multivariate Cox regression analysis was used to assess potential factors affecting breast cancer-specific survival (BCSS) and overall survival (OS) in patients with central breast cancer. Factors evaluated in the multivariate analysis model included surgery type, age at diagnosis, race, marital status, year at diagnosis, grade, T stage, N stage, ER status, PR status, and HER-2 status. To diminish the effects of baseline differences on outcome differences in the BCT and non-BCT groups, the propensity score matching (PSM) method was applied by matching each BCT case to non-BCT cases. They were exactly matched for the age, race, marital status, grade, T stage, N stage, ER status, PR status and HER-2 status. P < 0.05 was considered as an indicator of statistical significance. SPSS statistics (version 22, IBM, NY) was used to conduct all the above analyses.

## Results

### The trend of BCT and non-BCT among central breast cancer and relevant clinical characteristics

**Fig. 1:**
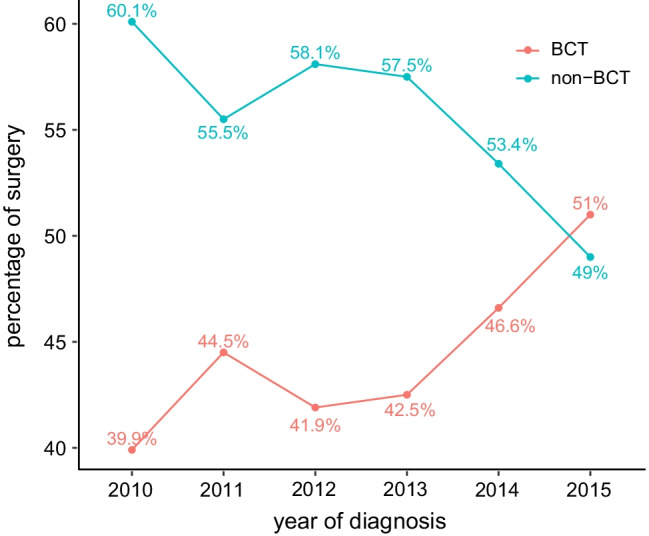
Proportion of patients with central breast cancer who underwent BCT and those who underwent non-BCT diagnosed between 2010 and 2015

From 2010 to 2015, a total of 8702 patients met our inclusion criteria and were included for analysis. The study consisted of 3870 (44.4%) patients with BCT and 4832 (55.6%) patients with non-BCT. The clinical characteristics of the BCT and non-BCT groups were summarized in Table 1. BCT was performed more frequently since 2010. Older patients, white patients, married patients, gradeII, early stage, T1 stage, N0 stage, ER positive, PR positive, HER-2 negative were more likely to receive BCT, and the proportion of those factors differed significantly between BCT and non-BCT group except for marital status. Comparing patients treated with non-BCT, patients initially treated with BCT were older at diagnosis (P < 0.001), have lower grade (P < 0.001), lower TNM stage (P < 0.001), lower T stage (P < 0.001), lower N stage (P < 0.001) and more likely to be ER positive at diagnosis (P < 0.001), PR positive at diagnosis (P < 0.001) and HER-2 negative at diagnosis (P < 0.001). They are also more likely to be of white race (P < 0.001). Figure 1 showed a trend of BCT for T1-4 central breast cancer and the BCT rate (51%) exceeded non-BCT in 2015.

**Table 1:** Comparison of patient and tumor characteristics between the BCT and non-BCT group

	BCT group		Non-BCT group		*P*-value
	No	%	No	%	
Years at diagnosis					< 0.001
2010	570	14.70	859	17.80	
2011	598	15.50	745	15.40	
2012	627	16.20	868	18.00	
2013	619	16.00	836	17.30	
2014	681	17.60	779	16.10	
2015	775	20.00	745	15.40	
Age					< 0.001
< 45	249	6.40	717	14.80	
45–59	1331	34.40	1861	38.50	
60–79	2290	59.20	2254	46.60	
Race					< 0.001
White	3165	81.80	3711	76.80	
Black	349	9.00	474	9.80	
Others	356	9.20	647	13.40	
Marital					0.439
Married	2370	61.20	2911	60.20	
Single	577	14.90	767	15.90	
Divorced	923	23.90	1154	23.90	
Grade					< 0.001
Grade I	1037	26.80	780	16.10	
Grade II	1908	49.30	2343	48.50	
Grade III	918	23.70	1692	35.00	
Grade IV	7	0.20	17	0.40	
Stage					< 0.001
Stage I	2218	57.30	1311	27.10	
Stage II	1439	37.20	2198	45.50	
Stage III	213	5.50	1323	27.40	
T stage					< 0.001
T1	2766	71.50	1924	39.80	
T2	971	25.10	1961	40.60	
T3	79	2.00	598	12.40	
T4	54	1.40	349	7.20	
N stage					< 0.001
N0	2810	72.60	2266	46.90	
N1	917	23.70	1687	34.90	
N2	107	2.80	560	11.60	
N3	36	0.90	319	6.60	
ER status					< 0.001
Negative	434	11.20	742	15.40	
Positive	3436	88.80	4090	84.60	
PR status					< 0.001
Negative	800	20.70	1263	26.10	
Positive	3070	79.30	3569	73.90	
HER-2 status					< 0.001
Negative	3350	86.60	3889	80.50	
Positive	520	13.40	943	19.50

### Predictive factors of BCT among central breast cancer

The results of multivariate logistic regression are reported in Table 2. Results confirmed that higher T stage (P < 0.001; T2: OR 0.447, 95% CI 0.402–0.496; T3: OR 0.152, 95% CI 0.118–0.195; T4: OR 0.182, 95%CI 0.134–0.247), higher N stage (P < 0.001; N1: OR 0.634, 95%CI 0.570–0.706; N2: OR 0.304, 95%CI 0.242–0.381; N3: OR 0.216, 95%CI 0.150–0.311), positive HER-2 status (P = 0.004; OR0.822 95%CI 0.719–0.940) and higher grade (P = 0.014; Grade II: OR 0.843, 95%CI 0.747–0.951; Grade III: OR 0.819, 95%CI 0.707–0.949) were independently associated with non-BCT. Other significant predictors of BCT include higher age (45–59 years: OR 2.026, 95% CI 1.706–2.405; 60–79 years: OR 2.581, 95% CI 2.182–3.053) and years at diagnosis (OR 1.076, 95% CI 1.048–1.106).

**Table 2:** Multivariate logistic regressions model for predictors of breast conserving therapy

Factor	OR	95%CI	*P*-value
Age			< 0.001
< 45	1	Reference	
45–59	2.026	1.706–2.405	< 0.001
60–79	2.581	2.182–3.053	< 0.001
Race			< 0.001
White	1	Reference	
Black	1.030	0.874–1.213	0.725
Others	0.680	0.585–0.79	< 0.001
Marital			0.059
Married	1	Reference	
Single	1.146	1.001–1.313	0.049
Divorced	0.952	0.850–1.067	0.4
Year of diagnosis	1.076	1.048–1.106	< 0.001
Grade			0.014
Grade I	1	Reference	
Grade II	0.843	0.747–0.951	0.005
Grade III	0.819	0.707–0.949	0.008
Grade IV	0.477	0.182–1.251	0.132
T stage			< 0.001
T1	1	Reference	
T2	0.447	0.402–0.496	< 0.001
T3	0.152	0.118–0.195	< 0.001
T4	0.182	0.134–0.247	< 0.001
N stage			< 0.001
N0	1	Reference	
N1	0.634	0.57–0.706	< 0.001
N2	0.304	0.242–0.381	< 0.001
N3	0.216	0.150–0.311	< 0.001
ER status			0.987
Negative	1	Reference	
Positive	1.002	0.829–1.209	0.987
PR status			0.082
Negative	1	Reference	
Positive	1.141	0.984–1.323	0.082
HER-2 status			0.004
Negative	1	Reference	
Positive	0.822	0.719–0.94	0.004

### Survival significance of BCT among central breast cancer

**Fig. 2:**
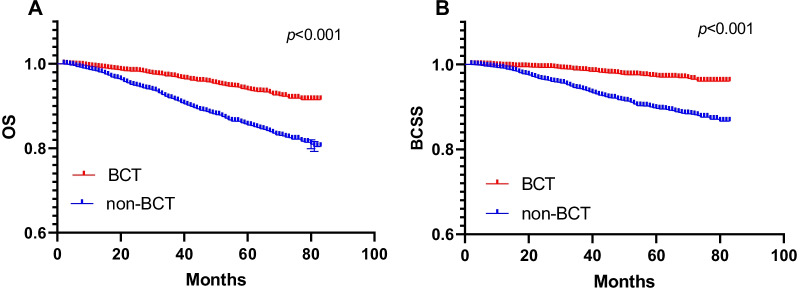
Kaplan–Meier survival curves of overall survival and breast cancer-specific survival stratified by BCT and non-BCT (**A**: OS; **B**: BCSS)

The Kaplan–Meier survival curve showed that BCT group had better OS and BCSS than non-BCT group (Fig. 2, both P < 0.001). For patients with central breast cancer, type of surgery, age, race, marital status, years at diagnosis, grade, T stage, N stage, ER status, PR status and HER-2 status were considered as potential prognostic variables and were included in the initial univariate and multivariate models. The results of the univariate analysis proportional hazard regression identified BCT significantly reduced overall death hazard (HR 0.396; 95%CT 0.332–0.473; P < 0.001) and breast-specific death hazard (HR 0.266; 95%CT 0.206–0.342; P < 0.001) (Tables 3, 4). And BCT still significantly reduced overall death hazard (HR 0.633; 95%CT 0.522–0.766; P < 0.001) and breast-specific death hazard (HR 0.570; 95%CT 0.435–0.746; P < 0.001) in the adjust multivariate Cox analysis. Other factors including age (P < 0.001), race (P < 0.001), marital status (P < 0.001), years at diagnosis (P = 0.038), grade (P < 0.001), T stage (P < 0.001), N stage (P < 0.001), ER status (P = 0.003), PR status (P < 0.001) and HER-2 status (P = 0.039) were identified as independent significant predictors of T1-4 central breast cancer overall mortality (OM), and race (P < 0.001), marital status (P = 0.007), grade (P < 0.001), T stage (P < 0.001), N stage (P < 0.001), ER status (P = 0.005), PR status (P < 0.001) and HER-2 status (P = 0.008) were identified as independent significant predictors of central breast cancer breast-specific mortality (BCSM).

**Table 3:** Univariable and multivariable models of overall mortality in central breast cancer patients

	Univariate analysis		Multivariate analysis	
	HR (95%CI)	*P*-value	HR (95%CI)	*P*-value
Surgery type		< 0.001		< 0.001
Non-BCT	Reference			
BCT	0.396 (0.332–0.473)	< 0.001	0.633 (0.522–0.766)	< 0.001
Age		< 0.001		< 0.001
< 45	Reference		Reference	
45–59	1.029 (0.769–1.378)	0.846	1.188 (0.885–1.595)	0.252
60–79	1.581 (1.201–2.080)	0.001	2.012 (1.518–2.668)	< 0.001
Race		< 0.001		< 0.001
White	Reference		Reference	
Black	1.922 (1.568–2.356)	< 0.001	1.509 (1.222–1.864)	< 0.001
Others	0.630 (0.466–0.851)	0.003	0.566 (0.418–0.767)	< 0.001
Marital		< 0.001		< 0.001
Married	Reference		Reference	
Single	1.596 (1.301–1.959)	< 0.001	1.366 (1.106–1.686)	0.004
Divorced	1.829 (1.544–2.166)	< 0.001	1.465 (1.231–1.742)	< 0.001
Year of diagnosis	0.929 (0.877–0.984)	0.012	0.941 (0.888–0.997)	0.038
Grade		< 0.001		< 0.001
Grade I	Reference		Reference	
Grade II	1.392 (1.081–1.793)	0.01	1.025 (0.792–1.326)	0.85
Grade III	3.189 (2.497–4.071)	< 0.001	1.581 (1.211–2.065)	0.001
Grade IV	4.950 (2.004–12.224)	0.001	2.438 (0.977–6.08)	0.056
T stage		< 0.001		< 0.001
T1	Reference		Reference	
T2	2.288 (1.906–2.747)	< 0.001	1.48 (1.214–1.805)	< 0.001
T3	4.055 (3.208–5.126)	< 0.001	1.947 (1.498–2.529)	< 0.001
T4	6.933 (5.452–8.817)	< 0.001	2.845 (2.169–3.731)	< 0.001
N stage		< 0.001		< 0.001
N0	Reference		Reference	
N1	1.83 (1.525–2.195)	< 0.001	1.461 (1.205–1.772)	< 0.001
N2	3.999 (3.214–4.976)	< 0.001	2.482 (1.956–3.149)	< 0.001
N3	6.087 (4.802–7.716)	< 0.001	3.180 (2.443–4.140)	< 0.001
ER status		< 0.001		0.003
Negative	Reference		Reference	
Positive	0.362 (0.307–0.427)	< 0.001	0.692 (0.544–0.880)	0.003
PR status		< 0.001		< 0.001
Negative	Reference		Reference	
Positive	0.407 (0.350–0.475)	< 0.001	0.666 (0.536–0.828)	< 0.001
HER-2 status		0.004		0.039
Negative	Reference		Reference	
Positive	1.318 (1.094–1.588)	0.004	0.813 (0.668–0.989)	0.039

**Table 4:** Univariable and multivariable models of breast cancer-specific mortality in central breast cancer patients

	Univariate analysis		Multivariate analysis	
	HR (95%CI)	P-value	HR (95%CI)	P-value
Surgery type		< 0.001		< 0.001
Non-BCT	Reference		Reference	
BCT	0.266 (0.206–0.342)	< 0.001	0.570 (0.435–0.746)	< 0.001
Age		< 0.001		0.894
< 45	Reference		Reference	
45–59	1.131 (0.843–1.518)	0.411	1.075 (0.79–1.463)	0.645
60–79	1.904 (1.437–2.524)	< 0.001	1.069 (0.785–1.455)	0.672
Race		< 0.001		< 0.001
White	Reference		Reference	
Black	1.505 (1.218–1.859)	< 0.001	1.473 (1.137–1.91)	0.003
Others	0.581 (0.429–0.787)	< 0.001	0.549 (0.374–0.806)	0.002
Marital		< 0.001		0.007
Married	Reference		Reference	
Single	1.355 (1.097–1.672)	0.005	1.244 (0.957–1.618)	0.103
Divorced	1.478 (1.243–1.758)	< 0.001	1.43 (1.141–1.792)	0.002
Year of diagnosis	0.935 (0.882–0.99)	0.022	0.949 (0.881–1.022)	0.167
Grade		< 0.001		< 0.001
Grade I	Reference		Reference	
Grade II	1.04 (0.804–1.346)	0.763	1.763 (1.109–2.803)	0.017
Grade III	1.612 (1.233–2.106)	0	3.159 (1.984–5.029)	< 0.001
Grade IV	2.439 (0.977–6.091)	0.056	4.019 (1.179–13.706)	0.026
T stage		< 0.001		< 0.001
T1	Reference		Reference	
T2	1.616 (1.329–1.966)	< 0.001	1.913 (1.441–2.54)	< 0.001
T3	2.241 (1.733–2.897)	< 0.001	2.798 (1.998–3.919)	< 0.001
T4	3.251 (2.487–4.25)	< 0.001	4.072 (2.868–5.782)	< 0.001
N stage		< 0.001		< 0.001
N0	Reference		Reference	
N1	1.532 (1.264–1.857)	< 0.001	1.907 (1.465–2.483)	< 0.001
N2	2.725 (2.151–3.452)	< 0.001	3.525 (2.599–4.781)	< 0.001
N3	3.518 (2.706–4.573)	< 0.001	4.546 (3.282–6.297)	< 0.001
ER status		0.003		0.005
Negative	Reference			
Positive	0.695 (0.546–0.885)	0.003	Reference	0.005
PR status		< 0.001		< 0.001
Negative	Reference		Reference	
Positive	0.664 (0.534–0.825)	< 0.001	0.519 (0.395–0.681)	< 0.001
HER-2 status		0.045		0.008
Negative	Reference		Reference	
Positive	0.818 (0.672–0.995)	0.045	0.723 (0.569–0.918)	0.008

### BCT as a prognostic factor for survival after propensity score matching

**Fig. 3:**
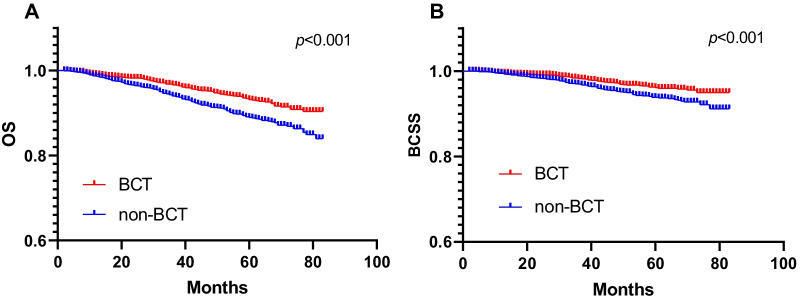
Kaplan–Meier survival curves of overall survival and breast cancer-specific survival stratified by BCT and non-BCT in matched case–control analysis (**A**: OS; **B**: BCSS)

**Fig. 4:**
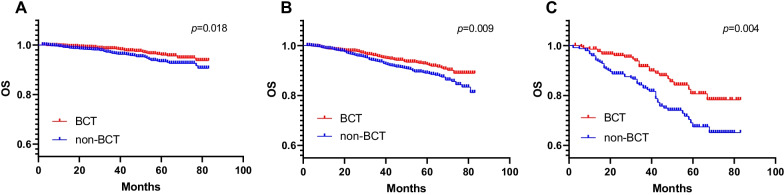
Kaplan–Meier survival curves of overall survival for BCT and non-BCT stratified by the stage in matched case–control analysis (**A** stage I; **B** stage II; **C** stage III)

**Fig. 5:**
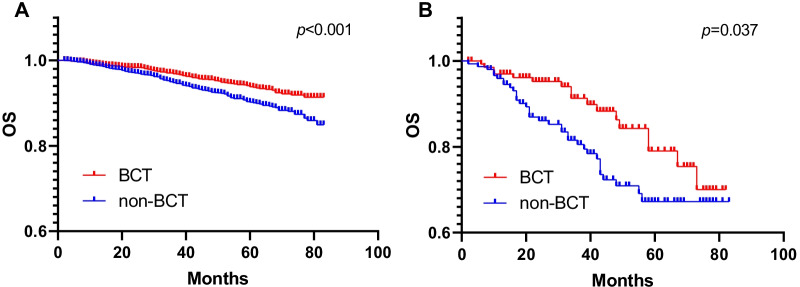
Kaplan–Meier survival curves of overall survival for BCT and non-BCT stratified by the T stage in matched case–control analysis (**A**: T1–2; **B**: T3–4)

To further corroborate the findings from univariable and multivariable proportional hazard regression, a propensity score-adjusted analysis was performed. A total of 2757 patients who underwent BCT were matched to 2757 patients who underwent non-BCT. Within the post-propensity cohort, there was no difference between both groups with regards to age (P = 0.114), race (P = 0.527), marital status (P = 0.287), grade (P = 0.669), T stage (P = 0.722), N stage (P = 0.547), ER status (P = 0.579), PR status (P = 0.409) and HER-2 status (P = 0.458) (Table 5). Using Kaplan–Meier survival estimates, BCT was associated with improved OS (P = 0.001) (Fig. 3) in the post-propensity cohort. In the subgroup analysis based on the post-propensity cohort. The beneficial impact of BCT on survival was additionally confirmed stratified for stage, and the P value were 0.018 for stage I, 0.009 for stage II, and 0.004 for stage III (Fig. 4). The BCT group had a higher OS compared with the non-BCT group in T1-2 (P < 0.001) and T3-4 (P = 0.037) (Fig. 5).

**Table 5:** Comparisons of clinicopathological characteristics between the BCT and non-BCT group in 1:1 matched case–control analysis

	Non-BCT		BCT		*P*-value
	No	%	No	%	
Year of diagnosis					< 0.001
2010	478	17.30	420	15.20	
2011	420	15.20	426	15.50	
2012	500	18.10	436	15.80	
2013	480	17.40	437	15.90	
2014	442	16.00	483	17.50	
2015	437	15.90	555	20.10	
Age					0.114
< 45	233	8.50	244	8.90	
45–59	1035	37.50	1101	39.90	
60–79	1489	54.00	1412	51.20	
Race					0.527
White	2202	79.90	2169	78.70	
Black	254	9.20	274	9.90	
Others	301	10.90	314	11.40	
Marital					0.287
Married	1713	62.10	1671	60.60	
Single	405	14.70	446	16.20	
Divorced	639	23.20	640	23.20	
Grade					0.669
Grade I	585	21.20	569	20.60	
Grade II	1360	49.30	1406	51.00	
Grade III	805	29.20	775	28.10	
Grade IV	7	0.30	7	0.30	
T stage					0.722
T1	1692	61.40	1676	60.80	
T2	918	33.30	948	34.40	
T3	85	3.10	79	2.90	
T4	62	2.20	54	2.00	
N stage					0.547
N0	1760	63.80	1799	65.30	
N1	843	30.60	815	29.60	
N2	108	3.90	107	3.90	
N3	46	1.70	36	1.30	
ER status					0.579
Negative	375	13.60	360	13.10	
Positive	2382	86.40	2397	86.90	
PR status					0.409
Negative	636	23.10	662	24.00	
Positive	2121	76.90	2095	76.00	
HER-2 status					0.458
Negative	2316	84.00	2337	84.80	
Positive	441	16.00	420	15.20

## Discussion

BCT involves excision of the tumor (lumpectomy) followed by adjuvant whole breast irradiation (WBI). In order to perform BCT, it must be possible to excise the tumor to negative margins with an acceptable cosmetic outcome, the patient must be able to receive radiotherapy, and the breast must be suitable for follow-up to allow prompt detection of local recurrence. Landmark trials have established that breast conservation therapy (BCT) and mastectomy offer equivalent survival and can be viewed as equivalent treatments in early stage breast cancer (ESBC) [17, 18]. Breast conserving therapy followed by radiotherapy allows patients to achieve esthetic outcomes, quality of life and preserve their breast without sacrificing oncologic outcome [1–3] and is considered as a safe treatment for early-stage breast cancer.

The term subareolar defined differently: Fowble et al. [7] and Haffty et al. [6] defined it as the area within 2 cm of the NAC, Haagensen shrank the distance to only 1 cm, and Simmons et al. [5] defined it as the area immediately beneath the areola. Central tumors usually refer to subareolar with some exceptions: only include NAC [19], tumors > 2 cm from areolar margin [7]. NAC malignant tumors included Paget disease, lymphoma and invasive and noninvasive breast cancers [20] and Paget disease were also a candidate for BCT [21]. In our study NAC account for 6.42% (559/8702) central and NAC patients, and the type of surgery did not correlated with location significantly (p = 0.692). But to date, the research on BCT of the NAC breast cancer is limited, so NAC breast cancer were included for further study. The early studies on the safety of BCT for CLBC [4, 13–16] or the comparation of oncological outcomes between BCT and non-BCT [7, 8] and the recent SEER based result [12] were all constrained to T1–2 stage. So in our study, T3–4 patients were included. Wang's study compared the safety of BCT versus mastectomy for CLBC [22]. But in our study, non-breast conserving patients included not only mastectomy, but also breast reconstruction.

Our result showed a trend of BCT for CLBC and it exceed non-BCT in 2015, and the proportion of BCT was similar to whole breast cancer reported in French (57%) and English (63%) [23]. We found a higher proportion of older age, single marital status, later years at diagnosis, lower grade, lower T stage, lower N stage, ER positive status, PR positive status and HER-2 negative status to receive BCT for CLBC and those factors were thought to be associated with favored outcome.

The young breast cancer always develops more aggressive tumors at diagnosis, like hormone receptor negative, higher grade, and HER-2 negative [24] and it is not contraindication for BCT for early stage patients. In our logistic analysis, we found that there is a significantly lower proportion of a young age (< 45 yeasts old) in BCT group (6.40%) compared with non-BCT group (14.8%). With the popularization of BRCA1/2 genetic testing and the maturity of breast reconstruction surgery, more and more young women are choosing breast reconstruction and contralateral prophylactic mastectomy [25, 26]. This may be why more young women are not opting for breast conserving surgery.

The evidence for breast conserving surgery has expanded with the availability of more drugs and improved efficacy of neoadjuvant therapy. Breast conserving surgery is not limited to early stage, such as T1–T2, but can be extended to T3–4. In our research, the OS rate of central breast cancer patents was higher with breast conserving surgery than with mastectomy, which was consistent with Zhang’s results [12]. However, our study demonstrates that T3–T4 and stage III patients receiving breast conserving therapy also had higher OS (P < 0.05).

And BCT significantly reduced overall death hazard (HR 0.633; 95%CT 0.522–0.766; P < 0.001) and breast-specific death hazard (HR 0.570; 95%CT 0.435–0.746; P < 0.001) in the adjust multivariate Cox analysis. When dug deeply, we found that there is a higher proportion of older age, single marital status, more recent years at diagnosis, lower grade, lower T stage, lower N stage, ER positive status, PR positive status and HER-2 negative status to receive BCT for CLBC and those factors were thought to be associated with favored survival outcome. To eliminate the effect of those confounders on prognosis analysis, propensity match score was used. Post-match cohort showed an improved survival in BCT compared with non-BCT in central and NAC tumors.

One limitation of breast conserving surgery for central breast cancer is postoperative aesthetics. In cases of tumor involvement of the nipple-areola complex, the surgeon may remove the nipple-areola complex to ensure a negative margin. This will bring great damage to postoperative breast aesthetics. Overall, nipple areola composite reconstruction will improve patient satisfaction and confidence. With the development of plastic surgery, a variety of methods of nipple areola composite reconstruction can be achieved, including tattooing, using synthetic materials, local flaps, and grafts [27–30]. This will make up for the shortcomings of breast conserving surgery in central breast cancer. Priya et al. demonstrated for patients with central tumor treated with neoadjuvant chemotherapy, many patients may have successfully converted to nipple-areola complex after reevaluation at the end of chemotherapy [31].

On the premise that the tumor safety and aesthetics can be achieved, breast conserving surgery for central breast cancer is a desirable option.

We recognize several limitations of this study. First of all, this study is a retrospective study with inherent flaws. Even though we use the PSM method, there will still be some biases. Secondly, because the patient's BRCA gene information is not available, it is impossible to evaluate its impact on the breast cancer surgery in the central region. Third, there is no information about postoperative complications, satisfaction and cosmetic results of breast conserving surgery in our study. Finally, the SEER database does not collect socioeconomic and baseline health information, which may be the relationship between surgical methods and survival. In the absence of prospective high-level evidence, our current large-sample retrospective study is of great significance to assess tumor safety, and more prospective studies are needed in the future.

## Conclusion

There is an increased incidence of BCT in patients with central breast cancer. Old age and low tumor malignancy were predictors of BCT. BCT is a safe and feasible surgical procedure for central breast cancer.

## Data Availability

These data were publicly available for use in accordance with a limited use agreement for SEER research data: Surveillance, Epidemiology, and End Results (SEER) Program (https://seer.cancer.gov) SEER*Stat Database.
